# The Female Perspective of Mating in *A. femoralis,* a Territorial Frog with Paternal Care – A Spatial and Genetic Analysis

**DOI:** 10.1371/journal.pone.0040237

**Published:** 2012-06-29

**Authors:** Eva Ringler, Max Ringler, Robert Jehle, Walter Hödl

**Affiliations:** 1 University of Vienna, Department of Evolutionary Biology, Vienna, Austria; 2 School of Environment and Life Sciences, Centre for Environmental Systems and Wildlife Research, University of Salford, Salford, United Kingdom; Université Paris 13, France

## Abstract

The adaptive significance of sequential polyandry is a challenging question in evolutionary and behavioral biology. Costs and benefits of different mating patterns are shaped by the spatial distribution of individuals and by genetic parameters such as the pairwise relatedness between potential mating partners. Thus, females should become less choosy as costs of mating and searching for mates increase. We used parentage assignments to investigate spatial and genetic patterns of mating across a natural population of the Neotropical frog *Allobates femoralis*, a species characterized by male territoriality and care and female iteroparity. There was no correlation between genetic and spatial distances between adult individuals across the population. In 72% of cases, females mated with males available within a radius of 20 m. Mean pairwise relatedness coefficients of successful reproducers did not differ from random mating but had a lower variance than expected by chance, suggesting maximal reproductive output at intermediate genetic divergence. We also found evidence for selection in favor of more heterozygous individuals between the embryo and adult stage. The level of sequential polyandry significantly increased with the number of spatially available males. Females that had more candidate males also produced more adult progeny. We hypothesize that the benefits associated with female multiple mating outweigh the costs of in- and outbreeding depression, and consequently precluded the evolution of ‘choosy’ mate selection in this species.

## Introduction

A key target in evolutionary biology is to determine the costs and benefits of specific mating patterns that have led to the wide range of animal mating systems. In resource-based mating systems, females are expected to select their mating partners because of direct benefits that are provided or accessible through males (such as food, shelter, parental care or protection). In nonresource-based systems, genetic quality in terms of ‘good’ or ‘compatible’ genes often constitutes an important factor regarding mate choice [1], [2]. Both systems can lead to the evolution of restrictive (i.e. choosy) mating, resulting in high reproductive skew in the non-choosing sex, but also to polyandry, a common mating system despite associated costs for females [3].

In cases where high levels of matings among close relatives result in a substantial reduction in overall population fitness due to inbreeding depression (for a review see [4]; see also [5]–[7]), a preference for unrelated partners in the choosing sex can constitute a strong selective benefit. At the other side of the spectrum, mating with very distantly related individuals can also have negative fitness consequences (outbreeding depression; [8], [9]). Accordingly, a preference for partners of intermediate relatedness has been found in fish [10], lizards [11], and birds [12]. However, in populations where genetic and spatial distance between individuals are positively correlated, a conflict between the acquisition of genetically optimal or preferable partners and the costs associated with searching for these mates might arise [11], [13].

Given that costs and benefits of choosiness are influenced by the number of spatially available candidate partners and the degree of variation in their genetic quality [14], females should become less choosy as costs of mating and/or searching for mates increase [15], [16], [17]. The evolution of polyandry has mainly been discussed for internally fertilizing species, where advantages of simultaneous polyandry arise from genetic benefits due to sperm competition or from increased fertilization success through post-copulatory mechanisms such as cryptic gamete choice [18]. Genetic benefits of polyandry in externally fertilizing species, with sequential polyandry in particular, have comparatively received rather little attention ([19], but see also [20], [21]).

Several studies have investigated spatial and genetic components of mate choice in mammals, birds, and fish (for reviews see [22]–[24]), but only very limited information is available on this subject for amphibians. Due to their complex reproductive behavior, dendrobatoid frogs are a particularly interesting taxon for studies on mate choice and reproductive success [25], [26]. The Neotropical frog *Allobates femoralis* Boulenger 1883 (Dendrobatoidea, Aromobatidae) is widespread across Amazonia where it forms disjunct local populations [27]. During the prolonged reproductive period, males are highly territorial with an average territory size of 11.01 m^2^ (range: 0.03–57.33 m^2^) [28], [29], [30]. They call from elevated structures to attract females as well as to announce territory occupancy to potential male competitors [31], and show highly aggressive behavior against calling intruders [32]. Female *A. femoralis* show site fidelity, but no aggressive behavior to individuals of either sex [29]. Pair formation, courtship, and mating take place in the male’s territory, where externally fertilized terrestrial clutches of approximately 20 eggs are laid in the leaf litter [33], [34], [35]. Tadpole transport is generally performed by males [33], [35]. A recent study showed that both sexes are highly iteroparous within a breeding season, and that territory possession but not territory size determines if males are considered as mating partners [30].

In the present study, we used spatial data and inferred genetic parentage of embryos obtained from clutches, as well as of adults of the following generation, to investigate patterns of parental relatedness in a natural *A. femoralis* population. This information allowed us (1) to investigate the prevalence of assortative mating by spatial proximity and/or genetic relatedness, (2) to reveal possible effects of in- and outbreeding depression, and (3) to relate female sequential polyandry and female reproductive output to the number of spatially available candidate males per female.

## Methods

Our study was approved by the scientific committee of the research station where fieldwork was conducted (http://www.nouragues.cnrs.fr/F-conseil.html). All necessary permissions for toe clipping and sampling of larvae were provided by the ‘Centre National de la Recherche Scientifique’ (CNRS, Permit Number: 12/05/2009) and by the ‘Direction Régionale de ĺEnvironment de Guyane’ (DIREN, Permit Number: arrêté n°/2010–015). All sampling was conducted in strict accordance with current French and EU law and followed the ASAB guidelines for the treatment of animals in behavioral research and teaching [36].

### Study Population

Our study population is located in a lowland rainforest near the field camp ‘Saut Pararé’ (4°02′ N, 52°41′ W) in the nature reserve ‘Les Nouragues’, French Guiana. Sampling took place between 15 January and 30 April 2008, and between 15 January and 15 March 2009, during the reproductive period of *A. femoralis* [37], [38]. The study plot was approximately 180 m×450 m in size, naturally delimited by a river, two streams, and an ascending ridge (for more details see [30]). In both years, surveys took place daily from 0900h to 1900h. We attempted total sampling of all male and female *A. femoralis* in the study plot in both years. Individuals were identified based on digital photographs of their ventral coloration patterns and sexed by the presence (males) or absence (female) of vocal sac folds. All spatial data on frogs and clutches were recorded in the field with the mobile GIS software ArcPad 7.0™ (ESRI) on pocket computers (Hewlett Packard iPaq™ HX4700) and further handled in ArcGIS™ 9.3 (ESRI). Capture-recapture studies in this and another *A. femoralis* population have shown that year-to-year survival is below 20%, resulting in rather discrete generations in consecutive years [29], [30].

### Tissue Sampling, Genotyping and Parentage Analysis

Detailed descriptions of the sampling procedures for adult individuals are given in [30], [39]. To obtain the embryos required for the present study, we sampled all clutches found within the study plot in 2008. We spent 10 min to search the leaf litter for clutches after every caught adult. Two embryos from every clutch were preserved in 96% ethanol as soon as the yolk sac was no longer visible (10–15 days of development [35]. Genomic DNA was isolated using a Proteinase K digestion followed by a standard phenol-chloroform protocol. PCR amplification of seven polymorphic microsatellite loci, genotyping and checking of genotyping errors followed the procedures described in [30].

Parentage data from adults, representing two successive generations (2008 and 2009), were already available from [30]. To infer the parentage of embryos sampled for the present study, we used an identical approach. We carried out all parentage assignments with the software COLONY v.2 [40], a likelihood-based method implementing a group-wise approach for sibship reconstruction to infer genealogies. Each embryo was treated without prior information about assumed full sib relationship of tadpoles from identical clutches.

### Relatedness Estimates

Pairwise relatedness coefficients *r* [41] for all possible male–female pairs in the parental generation (2008) were determined with KINGROUP [42]. This coefficient can be interpreted as a continuous measure of the overall genetic similarity between two individuals within a population. Values range from −1 to +1, with negative (positive) values indicating that two individuals have a lower (higher) probability of recent coalescence than random dyads within the population [41], [43], [44]. We used the simulation function in KINGROUP, based on the allele frequencies of our genotype data, to estimate the expected relatedness among 100 full siblings, 100 half siblings, and 100 ‘unrelated’ individuals in order to obtain reference intervals for closely related individuals. Pairwise relatedness is expected to average 0.5 for full sibs, 0.25 for half sibs, and zero as the population mean [44], [45]. The overall performance of this coefficient is expected to increase with sampling coverage, and was found to be accurate even when low numbers of loci with few alleles were used [44]. Given our sampling regime and sampling coverage for adult *A. femoralis* (see [30] for details), we assume this estimator to be very suitable for our analyses. In order to evaluate the congruence of the parentage and the relatedness estimates, we additionally calculated pairwise relatedness values between all adult individuals that were sampled in 2009. According to the sibship status inferred from the pedigree, we calculated mean parental relatedness of those individuals that were alleged full and half sibs, respectively, and compared the resulting measures to the values obtained by the KINGROUP simulations.

### Spatial Distribution of Pairwise Relatedness

**Figure 1:**
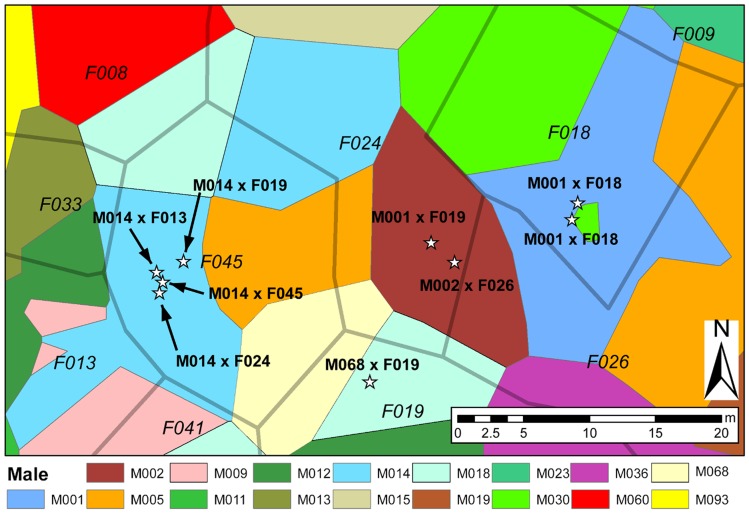
Spatial setup of Voronoi polygons and clutches. This figure displays an exemplary area of the study plot, showing overlapping Voronoi polygons of male and female *A. femoralis*, as well as the position and parental assignments of all clutches in this area. Voronoi polygons of males and females are indicated by colored areas and dark contours, respectively. According IDs of males are listed in the legend beside; IDs of females (in italics) are positioned inside their respective Voronoi areas.

To account for possible spatial effects on mating decisions we distinguished between three different sets of candidate males for each female: (1) all males inside the study plot (‘all ♂’); (2) males that were within 20 m of a female (‘♂ within 20 m’), a distance chosen based on observational data on the displacement of females from their resting sites during courtship (mean ± SD = 12.21±5.14 m, range 5.24–23.48 m; Ringler, Ursprung, and Hödl 2009); (3) all immediate neighbors to each female (‘♂ neighbors’) determined by creating Voronoi tessellations [46] based on all locations of males and females, respectively, using XTools Pro 7.1 [47] in ArcGIS™ 9.3 (ESRI). All areas that belonged to a certain individual were topologically joined to create a ‘Voronoi area’ for every individual. The Voronoi areas were created for males and females separately, and only those males whose Voronoi areas overlapped with that of a given female were assigned as direct neighbors to this female (see Figure 1). Spatial distances between individuals were calculated as the distance between the centroids of their Voronoi areas.

To identify a possible correlation of genetic relatedness and spatial distance between individuals, we compiled pairwise matrices of both values and tested for correlations between the two matrices using (partial) Mantel tests [48], [49]. This approach allowed us to consider all possible types of dyads (male–male, female–female, female–male) while controlling for the effects of the dyads out of interest (cf. [50]). All partial Mantel tests were calculated with the software zt [51], with 100,000 randomized permutations of the residual matrix.

### Analyses of Parental Relatedness

We used the embryo sample obtained for the present study as well as the cross-generational pedigree of adult individuals previously published in [30] to investigate patterns of relatedness across effective mating partners in 2008. Hence, we were able to identify successful mating events of the parental generation through clutch production, as well as through sexually mature offspring recorded in 2009. Initially, we conjointly analyzed mating information that was gained through both datasets (‘combined’), in order to use as much available mating information as possible. Since multiple adult offspring assigned to the same parent pair could have been the product of single or of multiple mating events, only binary mating information, e.g. whether a male had been identified as a mating partner of a given female or not, was used. Alternatively, we also analyzed the parentage information that was gained in each dataset (‘embryo’ and ‘pedigree’) separately to identify differences in parental relatedness across them. As single clutches could be treated as discrete mating events, information on repeated mating (i.e. multiple clutches) between identical partners in the parental generation was incorporated in tests involving only the embryo dataset.

To obtain means and unbiased variances of pairwise relatedness under random mating for each female, we generated 100,000 sets of mating partners randomly drawn from each of the three spatial sets of candidate males. The number of males per set equaled the number of effective mating partners of a given female (i.e. all male mating partners that were identified through offspring production). Randomizations without or with replacement were applied to generate values that correspond to the binary mating information or to allow for multiple mating between identical pairs. The permutations and calculations were performed in R version 2.12.2 (www.r-project.org; [52]).

To investigate the possibility of assortative mating we related parental relatedness with the number of clutches found, the number of embryos per clutch, and the number of adult progeny produced. We further tested whether the observed mean relatedness of females to their actual mating partners differed from the remainder of the candidate males, or from what could be expected under random mating with all candidate males. Additionally, we tested whether the observed variances in pairwise relatedness of females to their effective mating partners differed from variances expected under random mating. All analyses were performed in a paired design using the Wilcoxon signed rank test to account for the differences in pairwise relatedness between females and their respective candidate males. We additionally analyzed the data using Mann-Whitney *U* tests to distinguish between effects of individual female preferences for specific genotypes and general patterns across the whole population. We used the combined dataset for all tests, and the embryo dataset to also account for cases in which females chose specific males multiple times. All tests were performed across the three spatial categories of candidate males.

### The Relation between Sequential Polyandry and Reproductive Output

To investigate the effects of sequentially polyandrous mating in *A. femoralis* females, we first tested the relation between the number of effective mating partners per female and the number of candidate males available within 20 m. Under high sequential polyandry, these parameters should be strongly positively correlated. We further tested whether female reproductive output increased with the number of candidate males available within 20 m. As we considered the number of adult progeny produced to be the most significant measure of individual reproductive output, only this dataset was used for this analysis.

## Results

**Figure 2:**
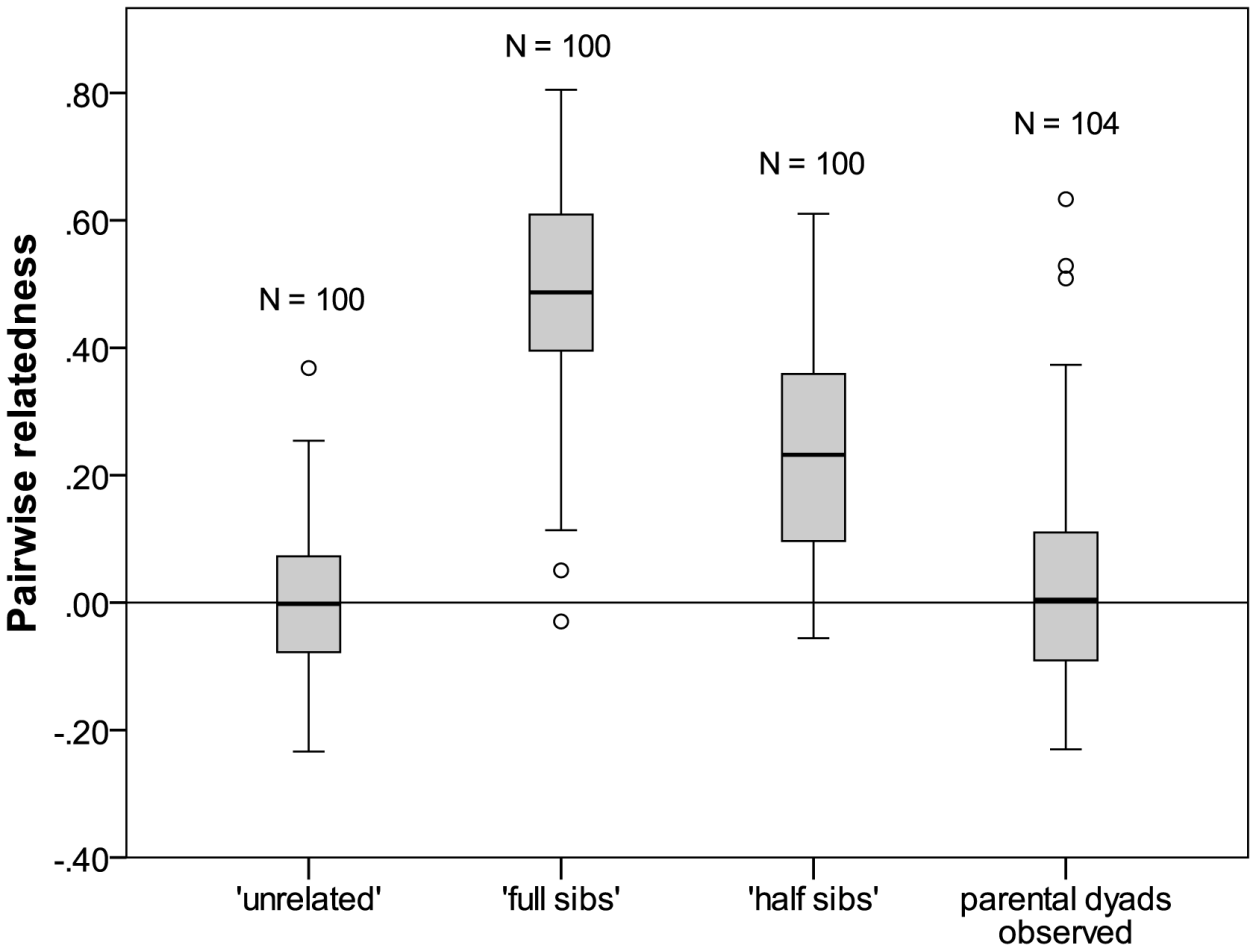
Pairwise relatedness of the KINGROUP simulations and of parental dyads observed in this study. The boxplots display the distribution of pairwise relatedness values in the KINGROUP simulations (‘unrelated’, ‘full sibs’, ‘half sibs’) and for parental dyads observed in this study.

In 2008, we recorded 139 adult *A. femoralis* (91 males and 48 females) and found 63 clutches across the study plot. Clutches contained on average 14.46 embryos (range: 3–22); the two embryos analyzed per clutch thus represent 17% of the embryos found. Embryos showed significantly lower heterozygosities than adult offspring (Wilcoxon signed rank test; *W* = −2.366; *P* = 0.018; Table 1), at non-significantly different numbers of alleles per locus (Wilcoxon signed rank test; *W* = −1.518; *P* = 0.129). The same pattern was revealed when only one random embryo per clutch was considered in these analyses (detailed data not shown). The KINGROUP simulations calculated an average pairwise relatedness of *r* ± SD = 0.489±0.162 for full siblings, *r* ± SD = 0.236±0.156 for half siblings, and *r* ± SD = 0.003±0.127 for ‘unrelated’ individuals (Figure 2). In comparison, the mean parental relatedness coefficients of identified full sibs and half sibs from 2009 were 0.41 (*N* = 44, SD = 0.18) and 0.21 (*N* = 89, SD = 0.14), respectively, well within the ranges obtained by the KINGROUP simulations.

**Table 1:** Number of alleles, expected and observed heterozygosities in the parental, the embryo, and the adult offspring sample.

	Parents*			Embryos			Adult progeny		
Locus	*N* = 139			*N* = 126			*N* = 128		
	*A*	*H*_O_	*H*_E_	*A*	*H*_O_	*H*_E_	*A*	*H*_O_	*H*_E_
*Afem03*	11	0.883	0.857	11	0.857	0.838	11	0.885	0.859
*Afem05*	17	0.555	0.613	16	0.437	0.48	14	0.63	0.625
*Afem09*	22	0.894	0.912	21	0.556	0.853	20	0.857	0.901
*Afem12*	16	0.905	0.872	16	0.881	0.854	17	0.935	0.882
*Afem13*	20	0.897	0.905	18	0.603	0.915	16	0.789	0.907
*Afem15*	21	0.917	0.908	18	0.643	0.891	18	0.839	0.891
*Afem16*	15	0.893	0.906	15	0.698	0.895	14	0.888	0.902

*A*, number of alleles; *H*_E_, expected heterozygosity; *H*_O_, observed heterozygosity.

* data from Ursprung et al. (2011a).

### Parentage Assignments

COLONY always assigned both embryos from a particular clutch to an identical parent pair. For 61 clutches (96.8%), paternity was assigned to the male that had been spatially closest at the presumed time it was sired (median father–clutch distance  = 0.97 m); the two remaining clutches had father–clutch distances of 3.61 m and 4.39 m, respectively. Maternity was in 89% of cases (56 clutches) assigned to a spatially close female (median mother–clutch distance  = 2.73 m); in 11% of cases, mothers had remained unsampled and were simulated by COLONY. Considering only mother–father dyads for which both parents were sampled, we identified 43 parental dyads based on the embryo dataset, and 66 parental pairs based on the analysis across the two adult generations (2008 and 2009, see [30]). Overall, the parentage analyses identified successful matings for 41 out of 48 (85.4%) females, and for 45 out of 91 (49.5%) males. On average, 64% of the mating partners per female were neighboring males, 72% were within 20 m distance (including neighboring males), and 28% were more than 20 m away. Four females mated exclusively with partners that were neither direct neighbors nor situated within 20 m distance. Twenty-one females mated exclusively with males within 20 m distance, and 18 of these mated exclusively with direct neighbors.

### Spatial Distribution of Pairwise Relatedness

There were no significant correlations between spatial distance and relatedness for the entirety of adult dyads in the parental generation (Mantel test; *r* = −0.007, *P* = 0.344), or when we separately analyzed the sexes (partial Mantel test; female–male: *r* = −0.007, *P* = 0.347, female–female: *r* = −0.007, *P* = 0.344, male–male: *r* = −0.006, *P* = 0.357). Mean pairwise relatedness for all 4368 possible male–female dyads within our population was −0.006 (s.d. = 0.153). In the whole population, 2% of all candidate males of any female were her full sibs (‘♂ within 20 m’: 2.1%; ‘♂ neighbors’: 2.4%), and 17% of candidate males were half sibs (‘♂ within 20 m’: 14%; ‘♂ neighbors’: 15%). According to the pairwise relatedness values of parental dyads, 2.4% of matings took place between full sibs, 15.2% between half sibs, and 82.4% between ‘unrelated’ individuals.

### Parental Relatedness

**Figure 3:**
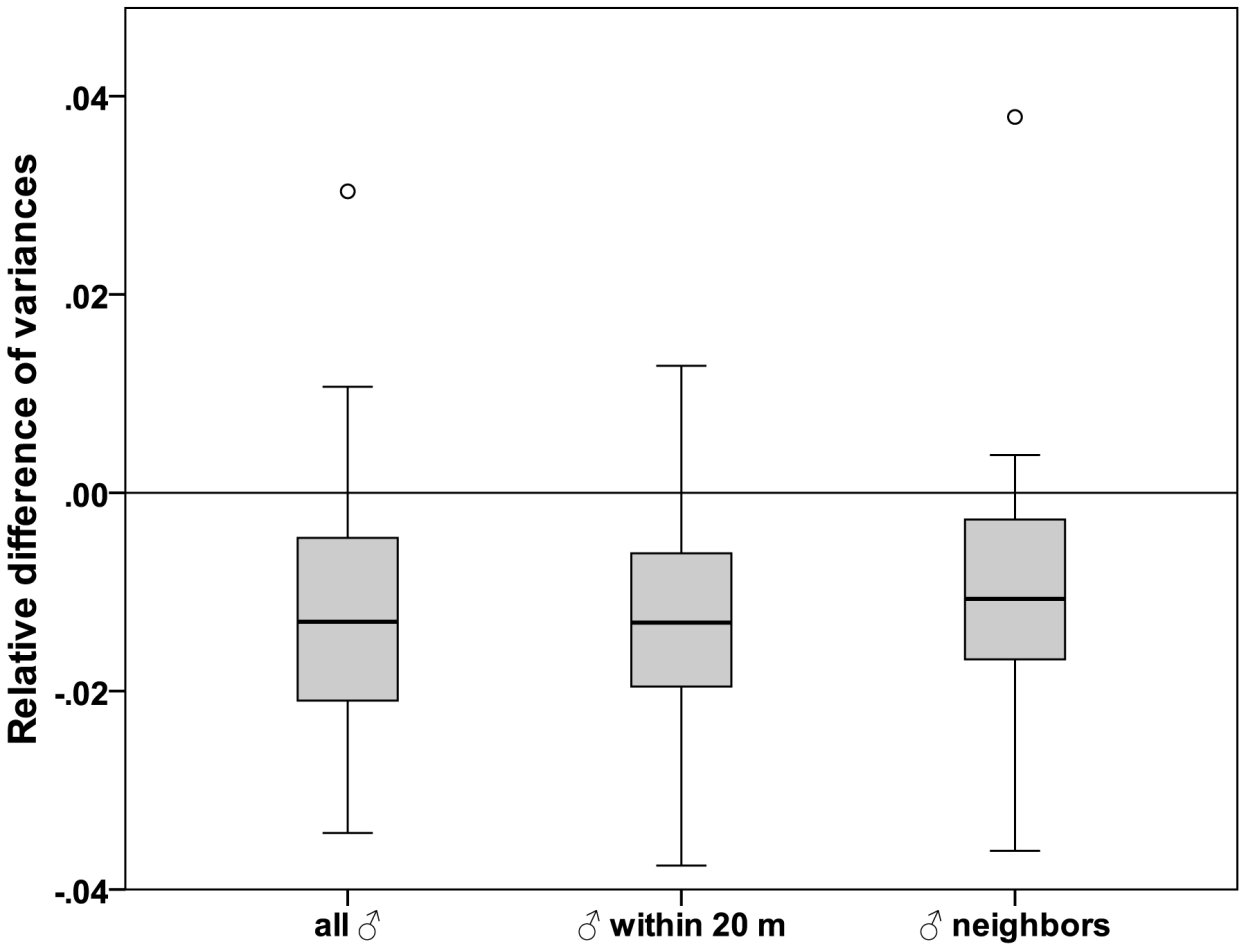
Deviation of the variance in pairwise relatedness from random mating. The figure indicates the deviation of the variances in pairwise relatedness of observed parental dyads from values expected under random mating for all three spatial categories of candidate males (all ♂, ♂ within 20 m, ♂ neighbors). Boxplots below zero indicate significant deviation from random expectations.

The variance in pairwise relatedness was significantly lower between each female and her effective mating partners than what would be expected under random mating (Table 2, Figure 3). However, mean pairwise relatedness of females to their mating partners did not differ from their relatedness to the remaining candidate males or from the mean relatedness to randomly selected males, regardless of statistical approach and dataset (Table 2). Pairwise relatedness of mother–father dyads was not linked to the number of clutches (Kruskal-Wallis test; *N* = 56, *H* = 2.496, df = 2, *P* = 0.287) and the number of adult offspring produced (*H* = 3.743, *N* = 66, df = 4, *P* = 0.443). The number of embryos per clutch was not linked to the pairwise relatedness of sires (*N* = 56, *r* = 0.044, *P* = 0.749).

**Table 2:** Analyses of parental relatedness.

	Mean		Variance	
	Combined	embryo	combined	embryo
all ♂	*N* = 41	*N* = 30	*N* = 27	*N* = 16
mate/non-mate	*W* = −0.03, *P* = 0.97	–	–	–
	*U* = 81, *P* = 0.75	–	–	–
mate/random	*W* = −0.06, *P* = 0.95	W = −0.59, P = 0.56	*W* = −3.39, *P* = 0.001	*W* = −3.52, *P*<0.001
	*U* = 94, *P* = 0.84	*U* = 37, *P* = 0.34	*U* = 132, *P*<0.001	*U* = 1, *P*<0.001
♂ within 20 m	*N* = 37	*N* = 29	*N* = 19	*N* = 16
mate/non-mate	*W* = −0.49, *P* = 0.62	–	–	–
	*U* = 84, *P* = 0.97	–	–	–
mate/random	*W* = −0.17, *P* = 0.86	*W* = −0.25, *P* = 0.804	*W* = 3.09, *P* = 0.002	*W* = −3.41, *P*<0.001
	*U* = 87, *P* = 0.99	*U* = 368, *P* = 0.54	*U* = 47, *P*<0.001	*U* = 23, *P*<0.001
♂ neighbors	*N* = 36	*N* = 27	*N* = 17	*N* = 15
mate/non-mate	*W* = −0.17, *P* = 0.86	–	–	–
	*U* = 82, *P* = 0.84	–	–	–
mate/random	*W* = −0.36, *P* = 0.72	*W* = −0.36, *P* = 0.79	*W* = −2.23, *P* = 0.03	*W* = −3.18, *P*<0.001
	*U* = 78, *P* = 0.55	*U* = 346, *P* = 0.93	*U* = 38, *P* = 0.006	*U* = 26, *P*<0.001

Wilcoxon signed rank test and Mann-Whitney *U* tests for differences in mean and variance in pairwise relatedness between females and their chosen mating partners and the remainder of candidate males (mate/non-mate), and randomly generated samples of equal size (mate/random), respectively, among all three candidate male categories.

### The Relation between Sequential Polyandry and Reproductive Output

**Figure 4:**
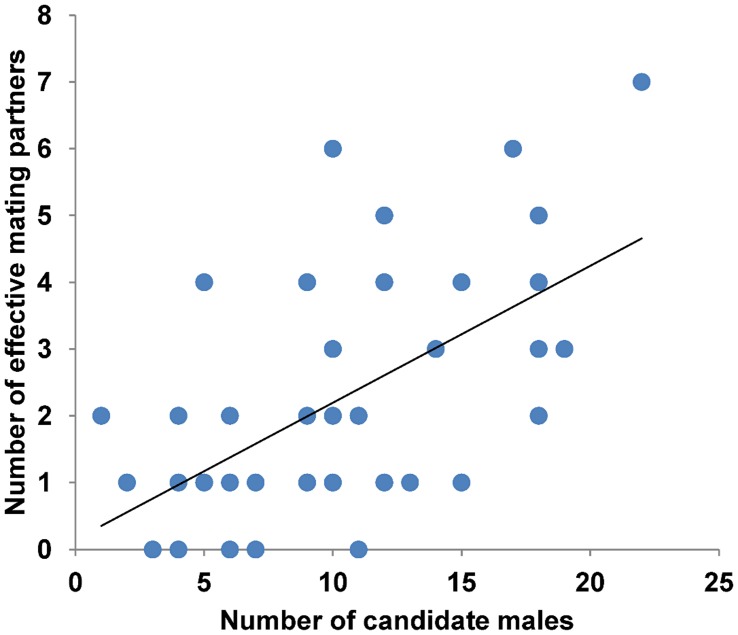
The relation between effective and available mating partners. The figure displays the number of effective mating partners per female in relation to the number of her candidate males available within 20 m.

**Figure 5:**
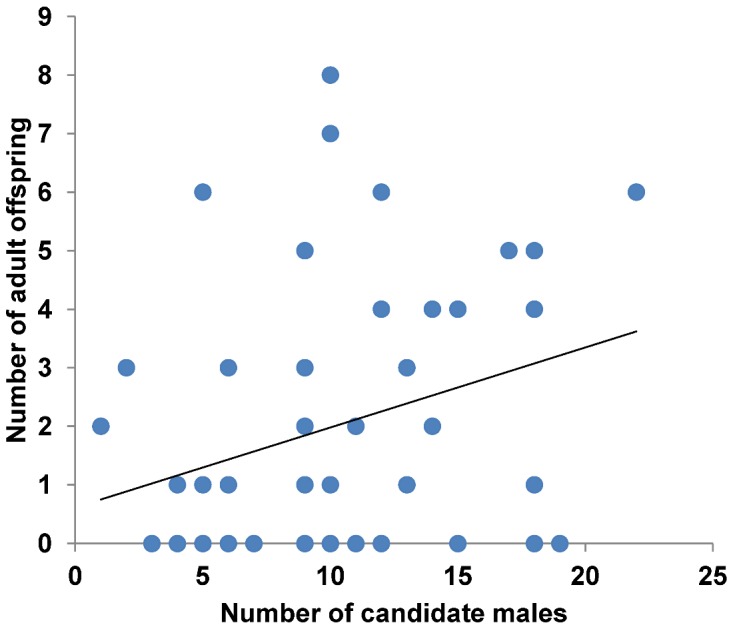
The relation between the number of candidate males and female reproductive output. The figure displays female reproductive output in terms of number of adult progeny produced in relation to the number of candidate males available.

The number of effective mating partners per female significantly increased with the number of candidate males within 20 m (*N* = 48, *r* = 0.586, *P*<0.001, Figure 4). Female reproductive output (number of adult progeny produced) significantly increased with the number of candidate males within 20 m (*N* = 48, *r* = 0.305, *P* = 0.035, Figure 5).

## Discussion

In the present study we investigated spatial and genetic patterns of reproductive success across a natural population of the Neotropical frog *A. femoralis*. The variance in pairwise relatedness of successful reproducers was smaller than expected from random mating, suggesting that reproductive success is highest between pairs of intermediate genetic divergence. Moreover, female reproductive output was strongly linked to the number of spatially available candidate males, which in turn was significantly correlated with the number of effective mating partners per female. Taken together, we hypothesize that the spatial distribution of males and females within the population as well as the benefits associated with sequential polyandry might have hindered the evolution of restrictive female choice in this species. Our results thus help to understand how sequential polyandry can evolve in species with nonresource-based mating systems and paternal care.

Research in the laboratory has provided remarkable insights in the interdependence of relatedness, mate choice, and reproductive success (for reviews see [8], [53]). Given that selective forces might have a different impact on populations in the wild than what has been observed under laboratory conditions [54], studies of species in their natural habitats are of particular value when investigating the adaptive significance of specific mating patterns on individual reproductive success.

We were able to identify mating success for 85.4% of females and 49.5% of males within the population. These values vastly exceed the findings from our previous study based on adult offspring alone (56.0% and 35.5%, respectively; [30]), likely due to mortality between the embryo and the adult stage. Females mainly mated with males within 20 m distance, which corroborates observational data on female movement during courtship [29]. For the four females that mated exclusively with males further than 20 m away we cannot rule out that they might have shifted their resting sites prior to, or after our field observations. COLONY always assigned identical parents to embryos from a particular clutch, thus providing no evidence for multiple paternities within clutches. Consequentially, all observations of polyandry in this study refer to sequentially polyandrous mating of *A. femoralis* females. We assume that multiple fertilizations are largely precluded by the elaborate courtship behavior and by the fact that mating takes place in the territories of males from where competitors are vigorously repelled [28], [32].

We found no correlation between genetic and spatial distance within the *A. femoralis* population, indicating a random to hyper-dispersed distribution of genotypes within the population. This pattern is likely caused by larval transport to water bodies and sexually unbiased dispersal of juveniles [29]. However, males situated far from a given female are presumably less likely to be chosen as mating partners. According to the spatial distribution of relatedness across all individuals, a rather low risk of inbreeding in the *A. femoralis* population can be assumed. As large groups of full sibs are precluded by the high polygynandry, as clutch size is relatively small compared to other anuran species, and as survival of embryos until adulthood is low, consequentially, the prevalence of full sib matings of about 2.4% is accordingly low. Previous studies also revealed that annual survival of adult *A. femoralis* is below 20% [29], [30]. As a consequence, most parents will have died before their progeny have become reproductively active, which reduces the likelihood of parent–offspring matings. As a result, it is unlikely that females could gain any genetic benefits from mating with spatially distant males, while energy expenditure and predation risk would likely increase with travel distance during mate search.

Mean pairwise relatedness of females to their mating partners did not differ from the mean relatedness to all other candidate males, or from what could be expected under random mating. This indicates that females show neither negative nor positive assortative mating with respect to male relatedness; the application of both, the Wilcoxon signed rank test and the Mann-Whitney *U* test, was necessary to distinguish between effects of individual female preferences for specific genotypes and general patterns of across the entire population. However, the variances in pairwise relatedness between parental pairs were significantly smaller than those expected under random mating, regardless of whether analyses accounted for female differences or not. These findings indicate a maximal reproductive output for mating partners of intermediate genomic divergence (cf. [10]), caused by either sexual (i.e. female choice) and/or natural selection (i.e. mortality).

Given that the proportion of observed full and half sib matings corresponded to the respective numbers of full and half sibs among the candidate males, we conclude that female *A. femoralis* do not avoid full and half sibs as mating partners. The absence of inbreeding avoidance or other forms of assortative mating by relatedness does not rule out a potential capability of *A. femoralis* females to recognize kin or genetic similarity. However, individual or kin recognition in amphibians is currently known only from larvae and early metamorphs, where it may have evolved due to the selective benefits of anti-predator behavior [55], [56] (but see also [57]). Females generally chose their partners within 20 m distance, and the number of effective mating partners increased significantly with the number of candidate males available within 20 m. Females seemed to mate randomly or evenly among all candidate males that are spatially close and we did not find any evidence for females being choosy.

Reproductive output in terms of clutches, embryos per clutch, or adult offspring produced did not significantly in- or decrease steadily with parental relatedness. Instead it were mating partners of intermediate genomic divergence that had maximal reproductive success, significantly higher than what could be expected under random mating, across all sets of candidate males. However, in fact our other findings indicate that females mate randomly or evenly with available males in spatial proximity. Consequentially, we argue that the observed maximum at intermediate relatedness is caused by a non-random survival of offspring with respect to the genetic divergence in parental genotypes. This is corroborated by the significantly lower heterozygosities in the embryo samples compared to the adult progeny, which indicates an increased mortality in inbred offspring. As maximal reproductive success was found at relatedness values close to the overall population mean, we assume that in- and outbreeding in the population results in similar levels of increased mortality (cf. [10], [11]). As selection may differentially impact specific larval stages [58], and because typically 80–95% of mortality in anuran amphibians occurs between hatching and metamorphosis [59], further studies under controlled conditions are needed to identify the effects of parental relatedness and individual heterozygosity on offspring survival at different life-history stages.

The number of effective mating partners per female was significantly related to the number of candidate males within 20 m. At the same time, females that had more males available also produced significantly more adult offspring. Accordingly, females with more mates produced significantly more offspring. Thus we assume that female *A. femoralis* actually make use of the opportunity for sequential polyandry and thereby increase their reproductive fitness without being choosy about their mates. Theoretical and empirical studies have repeatedly shown that sequential mating with multiple partners can compensate for negative effects of single mating decisions [1], [20], [60]. Sequential polyandry is particularly common in populations where the costs of in- and outbreeding avoidance, in terms of lost breeding opportunities, exceed the negative effects of in- and outbreeding (cf. [61], [62]). Alternatively, even incestuous matings can be preferable over mate rejection under certain circumstances [13], [63], [64]. In captivity, *A. femoralis* females were found to produce on average a clutch of 20 eggs every 8 days when well fed [35], and food was found not to be a limiting factor for this generalist feeder in natural populations [65]. We assume that *A. femoralis* females will ovulate and produce clutches at the maximum possible rate permitted by the actual food supply to maximize their reproductive success. Polygynandry in *A. femoralis* may thus act as a bet-hedging mechanism that insures against negative effects of single mating decisions [3], [66], especially with respect to the risks associated with paternal care, as females cannot influence their reproductive success after oviposition. Relatedness-based mate choice presumably did not evolve due to the random, highly dispersed distribution of related individuals across the population and the high costs of choosiness [64] (see also [67] for similar conclusions for *Physalaemus pustulosus*). We conclude that in- and outbreeding depression will have only marginal effects on the reproductive performance of the whole *A. femoralis* population, compared to other factors, and thus exert a negligible selective pressure to trigger the evolution of restrictive mate choice in this species.

However, the observed correlations between the number of partners, the number of mates, and the number of offspring could also have resulted from other factors such as habitat or female quality. In high quality habitats females might be able to produce more offspring, while simultaneously males could defend smaller territories that still provide sufficient resources/space. The resulting higher density of males would then lead to correlations similar to the ones we observed in our study. Although we cannot completely rule out such mutual, confounding effects, we do not assume that habitat quality plays a dominant role for the observed patterns of mating and reproductive success at the examined scale. Food as well as suitable calling and oviposition sites are abundant in tropical rainforests and therefore hardly are limiting factors for dendrobatoids [68]. Previous investigations did not find any relation between male territory size and individual reproductive output in *A. femoralis* [30]. Additionally, a recent study on the effects of reproductive resource supplementation found no effect of the proximity of water bodies used for tadpole deposition on the reproductive success of males and females in the same population (Ringler et al., in prep).

Likewise, the observed correlations could be a result of differential female quality. Females with a higher fecundity might attract more males to establish territories in their surroundings. In amphibians, the most commonly described surrogate for fecundity, allowing males to assess female quality, is female body size [69]. However, in a previous study we did not find a significant correlation between female body size and reproductive output [30]. Furthermore, so far neither the comprehensive observations from the field [29], [33], [34] nor from captivity [35] suggest any other mechanism how males could assess female fecundity beyond ‘counting’ the number of actually produced eggs. However, this mechanism seems equally unlikely, as males would need to frequently shift their territories during the breeding season, to optimize their location within the population as a reaction to experienced matings with low fecundity females. This hypothetical behavior is opposed by repeated observations of extremely high site fidelity of males throughout the whole breeding season [29], [33].

Future studies under controlled conditions are needed to reveal whether females choose their mating partners entirely randomly or if they are actively polyandrous by attempting to mate with as many of the available males as possible. Given the differences in demography, reproductive and spatial behavior among dendrobatoid species [70], this superfamily appears to be particularly well suited to investigate the evolution of different reproductive behaviors [26]. Comparative studies over various species with differing ecology are needed to gain insight not only into the reproductive dynamics in this taxon, but also increase our knowledge about factors that influence the evolution of different mating systems in general.

## References

[1] Colegrave N, Kotiaho JS, Tomkins JL (2002). Mate choice or polyandry: reconciling genetic compatibility and good genes sexual selection.. Evol Ecol Res.

[2] Neff BD Pitcher TE 2005 Genetic quality and sexual selection: an integrated framework for good genes and compatible genes. Mol Ecol 14: 19 38. Available: doi: 10.1111/j.1365-294X.2004.02395.x 15643948 10.1111/j.1365-294X.2004.02395.x

[3] Tregenza T Wedell N 2002 Polyandrous females avoid costs of inbreeding. Nature 415: 71 73. Available: doi:10.1038/415071a 11780118 10.1038/415071a

[4] Pusey A Wolf M 1996 Inbreeding avoidance in animals. Trends Ecol Evol 11: 201 206. Available: doi:10.1016/0169-5347(96)10028-8 21237809 10.1016/0169-5347(96)10028-8

[5] Rioux-Paquette E Festa-Bianchet M Coltman DW 2010 No inbreeding avoidance in an isolated population of bighorn sheep. Anim Behav 80: 865 871. Available: doi:10.1016/j.anbehav.2010.08.006

[6] Keller LF Waller DM 2002 Inbreeding effects in wild populations. Trends Ecol Evol 17: 230 241. Available: doi:10.1016/S0169-5347(02)02489-8

[7] Crnokrak P Barrett SCH 2002 Perspective: purging the genetic load: a review of the experimental evidence. Evolution 56: 2347 2358. Available: doi:10.1111/j.0014-3820.2002.tb00160.x 12583575 10.1111/j.0014-3820.2002.tb00160.x

[8] Tregenza T Wedell N 2000 Genetic compatibility, mate choice and patterns of parentage: invited review. Mol Ecol 9: 1013 1027. Available: doi:10.1046/j.1365-294x.2000.00964.x 10964221 10.1046/j.1365-294x.2000.00964.x

[9] Milinski M 2003 The function of mate choice in sticklebacks: optimizing Mhc genetics. J Fish Biol 63: 1 16. Available: doi:10.1111/j.1095-8649.2003.00215.x

[10] Neff BD 2004 Stabilizing selection on genomic divergence in a wild fish population. PNAS 101: 2381 2385. Available: doi:10.1073/pnas.0307522100 14983018 10.1073/pnas.0307522100 PMC356959

[11] Richard M Losdat S Lecomte J de Fraipont M Clobert J 2009 Optimal level of inbreeding in the common lizard. Proc R Soc Lond B 276: 2779 2786. Available: doi:10.1098/rspb.2009.0319 10.1098/rspb.2009.0319 PMC2839948 19419985

[12] Arct A Rutkowska J Martyka R Drobniak SM Cichoń M 2010 Kin recognition and adjustment of reproductive effort in zebra finches. Biol Lett 6: 762 764. Available: doi:10.1098/rsbl.2010.0417 20573618 10.1098/rsbl.2010.0417 PMC3001379

[13] Thünken T Bakker TCM Baldauf SA Kullmann H 2007 Active inbreeding in a cichlid fish and its adaptive significance. Curr Biol 17: 225 229. Available: doi:10.1016/j.cub.2006.11.053 17276915 10.1016/j.cub.2006.11.053

[14] Emlen ST Oring LW 1977 Ecology, sexual selection, and the evolution of mating systems. Science 197: 215 223. Available: doi:10.1126/science.327542 327542 10.1126/science.327542

[15] Bonachea LA Ryan MJ 2011 Localization error and search costs during mate choice in túngara frogs, *Physalaemus pustulosus.* Ethology 117: 56 62. Available: doi:10.1111/j.1439-0310.2010.01843.x

[16] Frère CH Krützen M Kopps AM Ward P Mann J 2010 Inbreeding tolerance and fitness costs in wild bottlenose dolphins. Proc R Soc Lond B 277: 2667 2673. Available: doi:10.1098/rspb.2010.0039 10.1098/rspb.2010.0039 PMC2982034 20392729

[17] Bleu J Bessa-Gomes C Laloi D 2012 Evolution of female choosiness and mating frequency: effects of mating cost, density and sex ratio. Anim Behav 83: 131 136. Available: doi:10.1016/j.anbehav.2011.10.017

[18] Simmons LW 2005 The evolution of polyandry: sperm competition, sperm selection, and offspring viability. Annu Rev Ecol Evol Syst 36: 125 146. Available: doi:10.1146/annurev.ecolsys.36.102403.112501

[19] Jennions MD Petrie M 2000 Why do females mate multiply? a review of the genetic benefits. Biol Rev 75: 21 64. Available: doi:10.1111/j.1469-185X.1999.tb00040.x 10740892 10.1017/s0006323199005423

[20] Byrne PG Roberts JD 2012 Evolutionary causes and consequences of sequential polyandry in anuran amphibians. Biol Rev 87: 209 228. Available: doi:10.1111/j.1469-185X.2011.00191.x 21740503 10.1111/j.1469-185X.2011.00191.x

[21] Evans JP Magurran AE 2000 Multiple benefits of multiple mating in guppies. PNAS 97: 10074 10076. Available: doi:10.1073/pnas.180207297 10954750 10.1073/pnas.180207297 PMC27698

[22] Clutton-Brock T McAuliffe K 2009 Female mate choice in mammals. Q Rev Biol 84: 3 27. Available: doi:10.1086/596461 19326786 10.1086/596461

[23] Kempenaers B 2007 Mate choice and genetic quality: a review of the heterozygosity theory. Adv Stud Behav 37: 189 278. Available: doi:10.1016/S0065-3454(07)37005-8

[24] Mays H Albrecht T Liu M Hill G 2008 Female choice for genetic complementarity in birds: a review. Genetica 134: 147 158. Available: doi:10.1007/s10709-007-9219-5 17973192 10.1007/s10709-007-9219-5

[25] Summers K, Amos W (1997). Behavioral, ecological, and molecular genetic analyses of reproductive strategies in the Amazonian dart-poison frog, *Dendrobates ventrimaculatus.*. Behav Ecol.

[26] Brown JL Morales V Summers K 2010 A key ecological trait drove the evolution of biparental care and monogamy in an amphibian. Am Nat 175: 436 446. Available: doi:10.1086/650727 20180700 10.1086/650727

[27] Amézquita A Lima AP Jehle R Castellanos L Ramos Ó 2009 Calls, colours, shape, and genes: a multi-trait approach to the study of geographic variation in the Amazonian frog *Allobates femoralis.* Biol J Linn Soc 98: 826 838. Available: doi:10.1111/j.1095-8312.2009.01324.x

[28] Ringler M, Ringler E, Magaña Mendoza D, Hödl W (2011). Intrusion Experiments to Measure Territory Size: Development of the Method, Tests Through Simulations, and Application in the Frog *Allobates femoralis*.. PLoS ONE.

[29] Ringler M Ursprung E Hödl W 2009 Site fidelity and patterns of short- and long-term movement in the brilliant-thighed poison frog *Allobates femoralis* (Aromobatidae). Behav Ecol Sociobiol 63: 1281 1293. Available: doi:10.1007/s00265-009-0793-7

[30] Ursprung E Ringler M Jehle R Hödl W 2011 Strong male/male competition allows for nonchoosy females: high levels of polygynandry in a territorial frog with paternal care. Mol Ecol 20: 1759 1771. Available: doi:10.1111/j.1365-294X.2011.05056.x 21410576 10.1111/j.1365-294X.2011.05056.x

[31] Hödl W Amézquita A Narins PM 2004 The role of call frequency and the auditory papillae in phonotactic behavior in male dart-poison frogs *Epipedobates femoralis* (Dendrobatidae). J Comp Physiol A 190: 823 829. Available: doi:10.1007/s00359-004-0536-1 10.1007/s00359-004-0536-1 15278399

[32] Narins PM Hödl W Grabul DS 2003 Bimodal signal requisite for agonistic behavior in a dart-poison frog, *Epipedobates femoralis.* PNAS 100: 577 580. Available: doi:10.1073/pnas.0237165100 12515862 10.1073/pnas.0237165100 PMC141038

[33] Roithmair ME (1994). Field studies on reproductive behaviour in two dart-poison frog species (*Epipedobates femoralis*, *Epipedobates trivittatus*) in Amazonian Peru.. Herpetol J.

[34] Montanarin A Kaefer IL Lima AP 2011 Courtship and mating behaviour of the brilliant-thighed frog *Allobates femoralis* from Central Amazonia: implications for the study of a species complex. Ethol Ecol Evol 23: 141 150. Available: doi:10.1080/03949370.2011.554884

[35] Weygold P (1980). Zur Fortpflanzungsbiologie von *Phyllobates femoralis* (Boulenger) im Terrarium.. Salamandra.

[36] ASAB 2006 Guidelines for the treatment of animals in behavioural research and teaching. Anim Behav 71: 245 253. Available: doi:10.1016/j.anbehav.2005.10.001 10.1006/anbe.1999.1349 10640387

[37] Born MG, Gaucher P, Bongers F, Charles-Dominique P, Forget P, Théry M (2001). Distribution and life histories of amphibians and reptiles..

[38] Gottsberger B Gruber E 2004 Temporal partitioning of reproductive activity in a neotropical anuran community. J Trop Ecol 20: 271 280. Available: doi:10.1017/S0266467403001172

[39] Ursprung E, Ringler M, Jehle R, Hödl W (2011). Toe regeneration in the neotropical frog *Allobates femoralis.*. Herpetol J.

[40] Wang J 2009 A new method for estimating effective population sizes from a single sample of multilocus genotypes. Mol Ecol 18: 2148 2164. Available: doi:10.1111/j.1365-294X.2009.04175.x 19389175 10.1111/j.1365-294X.2009.04175.x

[41] Queller DC, Goodnight KF (1989). Estimating relatedness using genetic markers.. Evolution.

[42] Konovalov DA Manning C Henshaw MT 2004 kingroup: a program for pedigree relationship reconstruction and kin group assignments using genetic markers. Mol Ecol Notes 4: 779–782. Available: doi:10.1111/j.1471-8286.2004.00796.x

[43] Blouin MS 2003 DNA-based methods for pedigree reconstruction and kinship analysis in natural populations. Trends Ecol Evol 18: 503 511. Available: doi:10.1016/S0169-5347(03)00225-8

[44] Konovalov DA Heg D 2008 A maximum-likelihood relatedness estimator allowing for negative relatedness values. Mol Ecol Resour 8: 256 263. Available: doi:10.1111/j.1471-8286.2007.01940.x 21585767 10.1111/j.1471-8286.2007.01940.x

[45] Blouin MS Parsons M Lacaille V Lotz S 1996 Use of microsatellite loci to classify individuals by relatedness. Mol Ecol 5: 393–401. Available: doi:10.1046/j.1365-294X.1996.00094.x 8688959 10.1111/j.1365-294x.1996.tb00329.x

[46] Voronoi G (1907). Nouvelles applications des paramètres continus à la théorie des formes quadratiques.. J Reine Angew Math.

[47] Data East (2003). XTools Pro for ArcGIS Desktop. 4.2.0 (Build 383): Data East, LLC.. http://www.xtoolspro.com/.

[48] Mantel N (1967). The detection of disease clustering and a generalized regression approach.. Cancer Res.

[49] Anderson MJ Legendre P 1999 An empirical comparison of permutation methods for tests of partial regression coefficients in a linear model. J Stat Comput Sim 62: 271 303. Available: doi:10.1080/00949659908811936

[50] Wagner AP Creel S Frank LG Kalinowski ST 2007 Patterns of relatedness and parentage in an asocial, polyandrous striped hyena population. Mol Ecol 16: 4356 4369. Available: doi:10.1111/j.1365-294X.2007.03470.x 17784926 10.1111/j.1365-294X.2007.03470.x

[51] Bonnet E, Van de Peer Y (2002). zt: a sofware tool for simple and partial mantel tests.. J Stat Softw.

[52] R Development Core Team (2010). R: a language and environment for statistical computing. Vienna Austria R Foundation for Statistical Computing.. http://www.r-project.org.

[53] Hettyey A Hegyi G Puurtinen M Hoi H Török J 2010 Mate choice for genetic benefits: time to put the pieces together. Ethology 116: 1 9. Available: doi:10.1111/j.1439-0310.2009.01704.x 21132114

[54] Halverson MA Skelly DK Caccone A 2006 Inbreeding linked to amphibian survival in the wild but not in the laboratory. J Hered 97: 499 507. Available: doi:10.1093/jhered/esl019 16957048 10.1093/jhered/esl019

[55] Cornell TJ Berven KA Gamboa GJ 1989 Kin recognition by tadpoles and froglets of the wood frog *Rana sylvatica.* Oecologia 78: 312 316. Available: doi:10.1007/BF00379103 28312575 10.1007/BF00379103

[56] Blaustein AR, Waldman B (1992). Kin recognition in anuran amphibians.. Anim Behav.

[57] Waldman B Rice JE Honeycutt RL 1992 Kin recognition and incest avoidance in toads. Am Zool 32: 18 30. Available: doi:10.1093/icb/32.1.18

[58] Ficetola GF Garner TWJ Wang J de Bernardi F 2011 Rapid selection against inbreeding in a wild population of a rare frog. Evol Appl 4: 30 38. Available: doi:10.1111/j.1752-4571.2010.00130.x 25567951 10.1111/j.1752-4571.2010.00130.x PMC3352519

[59] Vonesh J de La Cruz O 2002 Complex life cycles and density dependence: assessing the contribution of egg mortality to amphibian declines. Oecologia 133: 325 333. Available: doi:10.1007/s00442-002-1039-9 28466219 10.1007/s00442-002-1039-9

[60] Roberts SC Gosling LM 2003 Genetic similarity and quality interact in mate choice decisions by female mice. Nat Genet 35: 103 106. Available: doi:10.1038/ng1231 12937417 10.1038/ng1231

[61] Pärt T (1996). Problems with testing inbreeding avoidance: the case of the collared flycatcher.. Evolution.

[62] Jamieson IG Taylor SS Tracy LN Kokko H Armstrong DP 2009 Why some species of birds do not avoid inbreeding: insights from New Zealand robins and saddlebacks. Behav Ecol 20: 575 584. Available: doi:10.1093/beheco/arp034

[63] Kokko H, Ekman J (2002). Delayed dispersal as a route to breeding: territorial inheritance, safe havens, and ecological constraints.. Am Nat.

[64] Kokko H Ots I 2006 When not to avoid inbreeding. Evolution 60: 467 475. Available: doi:10.1554/05-613.1 16637492

[65] Toft CA (1980). Feeding ecology of thirteen syntopic species of anurans in a seasonal tropical environment.. Oecologia.

[66] Byrne PG Keogh JS 2009 Extreme sequential polyandry insures against nest failure in a frog. Proc R Soc Lond B 276: 115 120. Available: doi:10.1098/rspb.2008.0794 10.1098/rspb.2008.0794 PMC2614246 18782745

[67] Lampert KP Bernal XE Rand AS Mueller U Ryan MJ 2006 No evidence for female mate choice based on genetic similarity in the túngara frog *Physalaemus pustulosus.* Behav Ecol Sociobiol 59: 796 804. Available: doi:10.1007/s00265-005-0125-5

[68] Pröhl H (2005). Territorial Behavior in Dendrobatid Frogs.. J Herpetol.

[69] Halliday TH, Verrell PA (1988). Body Size and Age in Amphibians and Reptiles.. J Herpetol.

[70] Lötters S, Jungfer K, Henkel FW, Schmidt W (2007). Poison frogs. Biology, species & captive husbandry. Frankfurt am Main, Germany: Edition Chimaira.. 668 p.

